# Conjugates of methylene blue with γ-carboline derivatives as new multifunctional agents for the treatment of neurodegenerative diseases

**DOI:** 10.1038/s41598-019-41272-4

**Published:** 2019-03-19

**Authors:** Sergey O. Bachurin, Galina F. Makhaeva, Elena F. Shevtsova, Natalia P. Boltneva, Nadezhda V. Kovaleva, Sofya V. Lushchekina, Elena V. Rudakova, Ludmila G. Dubova, Daria V. Vinogradova, Vladimir B. Sokolov, Alexey Yu. Aksinenko, Vladimir P. Fisenko, Rudy J. Richardson, Gjumrakch Aliev

**Affiliations:** 1 Institute of Physiologically Active Compounds Russian Academy of Sciences, Chernogolovka, 142432 Russia; 2 Emanuel Institute of Biochemical Physics, Russian Academy of Sciences, Moscow, 119334 Russia; 3 I.M. Sechenov First Moscow State Medical University (Sechenov University), Moscow, 119991 Russia; 4 Department of Environmental Health Sciences, University of Michigan, Ann Arbor, MI 48109 USA; 5 Department of Neurology, University of Michigan, Ann Arbor, MI 48109 USA; 6 Center for Computational Medicine and Bioinformatics, University of Michigan, Ann Arbor, MI 48109 USA; 7 GALLY International Biomedical Research Consulting LLC., San Antonio, TX 78229 USA; 8 School of Health Science and Healthcare Administration, University of Atlanta, Johns Creek, GA 30097 USA

## Abstract

We studied the inhibitory activity of methylene blue (MB) γ-carbolines (gC) conjugates (MB-gCs) against human erythrocyte acetylcholinesterase (AChE), equine serum butyrylcholinesterase (BChE), and a structurally related enzyme, porcine liver carboxylesterase (CaE). In addition, we determined the ability of MB-gCs to bind to the peripheral anionic site (PAS) of *Electrophorus electricus* AChE (*Ee*AChE) and competitively displace propidium iodide from this site. Moreover, we examined the ability of MB-gCs to scavenge free radicals as well as their influence on mitochondrial potential and iron-induced lipid peroxidation. We found that MB-gCs effectively inhibited AChE and BChE with IC_50_ values in the range 1.73–10.5 μM and exhibited low potencies against CaE (9.8–26% inhibition at 20 μM). Kinetic studies showed that MB-gCs were mixed-type reversible inhibitors of both cholinesterases. Molecular docking results showed that the MB-gCs could bind both to the catalytic active site and to the PAS of human AChE and BChE. Accordingly, MB-gCs effectively displaced propidium from the peripheral anionic site of *Ee*AChE. In addition, MB-gCs were extremely active in both radical scavenging tests. Quantum mechanical DFT calculations suggested that free radical scavenging was likely mediated by the sulfur atom in the MB fragment. Furthermore, the MB-gCs, in like manner to MB, can restore mitochondrial membrane potential after depolarization with rotenone. Moreover, MB-gCs possess strong antioxidant properties, preventing iron-induced lipid peroxidation in mitochondria. Overall, the results indicate that MB-gCs are promising candidates for further optimization as multitarget therapeutic agents for neurodegenerative diseases.

## Introduction

Alzheimer’s disease (AD) is one of the most common neurodegenerative diseases. It is characterized by progressive loss of memory and higher cortical functions leading to total cognitive and intellectual decline^1^.

The multifactorial nature of AD is now generally accepted^2^. Key aspects of AD pathogenesis are cholinergic and glutamatergic mediator systems dysfunction, aberrant protein deposition (β-amyloid and tau protein), oxidative stress^1^, together with impairment of mitochondrial function (impairments of oxidative phosphorylation, decreased calcium retention capacity, and increased vulnerability to induction of the mitochondrial permeability transition)^3^.

The first drug approved for AD treatment was the acetylcholinesterase (AChE) inhibitor tacrine, with potent effects in restoring cholinergic deficit^4^. Currently, the most common therapeutic agents for AD are inhibitors of cholinesterases (mostly AChE): donepezil (Aricept), rivastigmine (Exelon), and galantamine. In addition, the low-affinity non-competitive NMDA receptor antagonist memantine is prescribed for patients with moderate to severe AD who do not tolerate cholinesterase inhibitors well^5^. However, although the above-mentioned drugs may attenuate symptoms, they do not stop the initiation or progression of AD.

It is now widely believed that soluble oligomeric forms of amyloid-β aggregates containing 40–42 amino acid residues (Aβ 40–42) and preceding senile plaques formation are neurotoxic. They are assumed to disrupt mitochondrial functions, induce apoptosis, and regulate stress-activated protein^6^. Drugs decreasing brain Aβ levels by either slowing formation or enhancing clearance are presumed to be able to stop or even reverse AD.

Apart from its classical acetylcholine hydrolysis function, AChE reportedly has pro-aggregator properties for Aβ^7^. AChE plays an important role in the processing of Aβ through the interaction of its peripheral anionic site (PAS) with soluble amyloid-β peptides to promote their aggregation^8–10^. Based on these facts, drugs with such dual capabilities (i.e., inhibition of AChE catalytic activity and inhibition of AChE-induced Aβ aggregation) have been a subject of intensive research^11^. It is therefore reasonable to expect that these kinds of agents could simultaneously enhance cognition and engender neuroprotection^12,13^.

Oxidative stress is one of the important factors negatively affecting neuronal function in the brain. It is characterized by an imbalance between reactive oxygen species production and their removal by various mechanisms of the antioxidant systems. It should be noted that brain is more vulnerable to oxidative stress^14–16^ than any other tissue. Moreover, the efficacy of the brain antioxidant system progressively declines with aging. Notably, this decline is more dramatic in AD brain. Accordingly, it would be reasonable to use antioxidants in AD therapy^14,17^, and the development of cholinesterase inhibitors with additional antioxidant properties is a present-day trend in the search for new effective treatments for AD^18–20^.

Whereas chronological aging is the main risk factor in sporadic AD, increased susceptibility to the mitochondrial permeability transition, defects in energy metabolism, and impairment of other mitochondrial functions are considered among the earliest manifestations of AD pathogenesis. Mitochondria play an important role in production of amyloid peptides and ROS and simultaneously mitochondria are the targets of their toxic action^21^. Thus, the search for effective mitoprotection agents has great potential for uncovering compounds that could be useful in AD therapeutics^3^.

**Figure 1:**
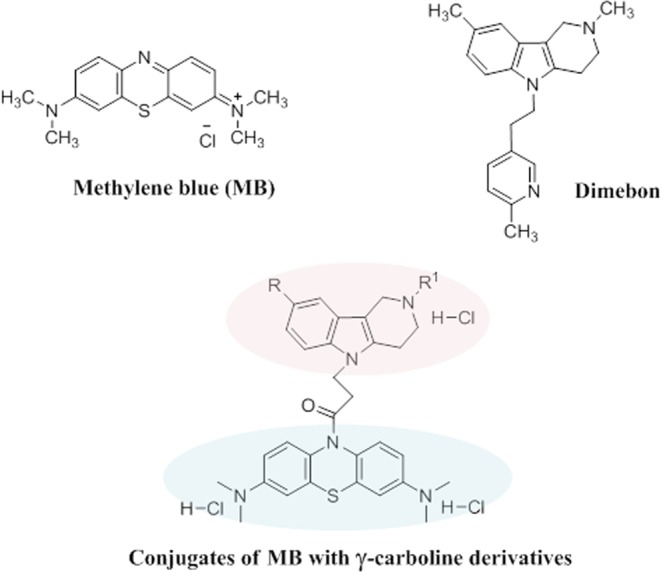
Structures of Methylene blue, Dimebon, and the studied conjugates of MB and γ-carboline derivatives **1–7** (R = H, CH_3_, F; R^1^ = alkyl).

Considering the multiplicity of biological pathways involved in AD pathogenesis and progression, the discovery and development of multifunctional, multi-targeted agents with complex actions on a combination of biological targets involved in AD pathogenesis is both an exceptionally challenging and promising strategy^22–30^. In contrast to the polypharmacy approach, a single drug molecule that could simultaneously attenuate multiple pathogenic pathways would simplify the tasks of optimizing pharmacokinetics and reducing toxicity. Thus, in the present study, we used methylene blue (MB) and the γ-carboline fragment of Dimebon as initial pharmacophores to design hybrid multifunctional molecules with the desired characteristics for potential AD therapeutics (Fig. 1).

MB is a member of the phenothiazine family. It has a wide range of therapeutic applications including treatment of methemoglobinemia and ifosfamide-induced encephalopathy. Recent studies reported that MB possesses a high potential for treating Alzheimer’s disease based on its memory improvement and neuroprotective properties^31–34^. In rodents, MB was shown to improve memory in normal brain as well as in various animal models of neurodegenerative diseases including AD^35^ and Huntington’s disease^36^. *In vitro* studies support the contention that MB inhibits the formation of β-amyloid oligomers by promoting fibril formation^32,37,38^ and Huntington protein aggregation^36^. It also exerts an anti-tau aggregation effect^32,39^. In addition, the background for our selection of this pharmacophore included previous observations that phenothiazine derivatives including MB can effectively inhibit cholinesterases^40–42^.

Due to both cationic and lipophilic properties, MB easily crosses the blood brain barrier^31^, binds to mitochondrial membranes, and diffuses into the mitochondrial matrix, where at low concentrations it enters into a redox equilibrium with the enzymes of the electron transport chain. In so doing, MB enhances mitochondrial respiration, thereby increasing ATP production and oxygen consumption. Thus, MB is a potent redox agent with high bioavailability to mitochondria^33,43^ that decreases mitochondrial ROS formation, consequently delaying cellular senescence. MB can decrease oxidative damage in pro-oxidant conditions and thus prevent nerve cell death induced by oxidative stress^44^ and inhibit rotenone-induced lipid peroxidation^45^. MB also diminishes oxidative stress-induced AD-like tau and β-amyloid aggregation *in vitro*^38,46^.

γ-Carboline derivatives (gCs) have attracted close attention in recent years as new-generation agents for the treatment of various neurodegenerative diseases including AD^47–50^. A well-known representative of this series of compounds is the antihistamine drug Dimebon (Latreperdine) (Fig. 1), which possesses a broad spectrum of pharmacological activities^51,52^. In particular, this drug improves cognitive function^48,53–55^ and exhibits neuroprotective, antidepressant, and geroprotective actions^52,54,56^. Dimebon successfully passed phase II clinical trials^48^. Unfortunately, these promising findings could not be replicated during phase III trials conducted in multiple centers involving heterogeneous populations including neurological conditions other than AD^57,58^.

Recent studies showed that Dimebon and MB are able to protect neurons in different models of neurodegeneration^43,52,58–60^. Moreover, significant protective effects were observed in an *in vitro* model of ALS when both compounds were administered simultaneously^61^. In this context, we recently synthesized a group of original compounds that combine MB and the gC fragment of Dimebon in one molecule (Fig. 1) as a novel approach to the development of multitarget disease-modifying agents. In addition, we studied their binding to different NMDA receptor modulatory sites^62^.

Here, we have investigated the action of the novel MB-gCs **1–7** on enzyme targets of the cholinergic nervous system using as surrogates human erythrocyte acetylcholinesterase (EC 3.1.1.7, AChE) and equine serum butyrylcholinesterase (EC 3.1.1.8, BChE), along with a structurally related enzyme, porcine liver carboxylesterase (EC 3.1.1.1, CaE). We also studied the ability of MB-gCs to bind to the PAS of AChE from *Electrophorus electricus* (*Ee*AChE) and competitively displace propidium iodide from this site. Enzyme kinetics was used to ascertain the mechanism of inhibition and molecular docking was employed to explain it. In addition, we measured the radical-scavenging activity of MB-gCs by means of the ABTS and ORAC-FL assays. The antioxidant effect of MB-gCs was evaluated by measuring iron-induced lipid peroxidation (LP). We also assessed their effect on mitochondrial potential and calcium-induced mitochondrial depolarization. MB, reduced MB (МВН_2_, leucomethylene blue) and Dimebon were used as reference compounds in all experiments.

## Results

### Inhibition of AChE, BChE and CaE by MB-gCs and kinetic analysis

All conjugates of MB and gCs were evaluated for their ability to inhibit AChE, BChE, and CaE. AChE from human erythrocytes, BChE from equine serum, and CaE from porcine liver were used. It was previously shown that the two last-mentioned enzymes have high levels of identity with the corresponding human enzymes^63,64^.

The inhibitory ability was characterized as % inhibition at 20 μM or by the IC_50_–the inhibitor concentration required to reduce the enzyme activity by 50%. The results summarized in Table 1 show that the studied MB-gCs very weakly inhibit CaE and show rather high inhibitory activity against AChE and BChE.

**Table 1:** Inhibitory activity of MB-gCs toward AChE, BChE, and CaE.

Compounds			IC_50_ (µM) or % inhibition at 20 µM		
No	R	R^1^	AChE	BChE	CaE
**1**	H	CH_3_	3.17 ± 0.13	5.94 ± 0.55	>20 (21.5 ± 2.3%)
**2**	H	C_2_H_5_	2.95 ± 0.07	2.53 ± 0.22	>20 (18.1 ± 1.7%)
**3**	CH_3_	CH_3_	1.73 ± 0.11	1.82 ± 0.14	>20 (26.0 ± 2.7%)
**4**	CH_3_	C_2_H_5_	4.03 ± 0.36	17.9 ± 1.4	>20 (15.7 ± 1.8%)
**5**	CH_3_	*n*-C_3_H_7_	3.95 ± 0.21	8.42 ± 0.83	>20 (9.8 ± 1.4%)
**6**	CH_3_	*i*-C_3_H_7_	7.37 ± 0.42	0.97 ± 0.05	>20 (19.5 ± 1.8%)
**7**	F	CH_3_	6.86 ± 0.32	10.5 ± 0.9	>20 (13.8 ± 1.6%)
MB			1.21 ± 0.09	11.1 ± 0.1	>20 (12.3 ± 1.5%)
МBН_2_ (Leuco form)			1.76 ± 0.09	10.7 ± 0.2	>20 (17.2 ± 1.8%)
Dimebon			36.3 ± 3.59	5.76 ± 0.51	n.а.

n.a. – not active at 20 µM.

Data are expressed as mean ± SEM (*n* ≥ 3).

Data expressed as % correspond to % inhibition at 20 μM.

Data shown without units of measurement are IC_50_ values in μM.

The compounds inhibited AChE and BChE in the micromolar range without clear selectivity. All of the conjugates were somewhat less efficient AChE inhibitors than MB itself, while for most of them, the potency against BChE was higher than for MB and comparable or higher than that for Dimebon. Compound **3** (R = R^1^ = CH_3_) had the highest activity against AChE, and compound **6** (R = CH_3_, R^1^ = i-C_3_H_7_) has the highest activity against BChE.

**Figure 2:**
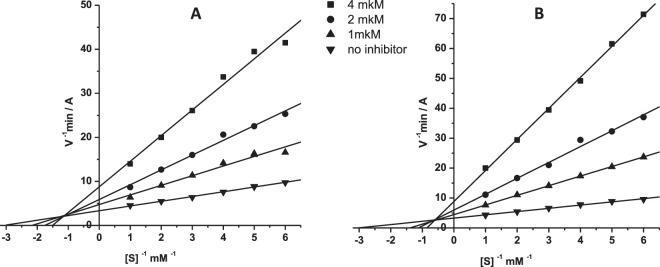
Steady state inhibition of AChE (**A**) and BChE (**B**) by compound **3**. Lineweaver-Burk reciprocal plots of initial velocity and substrate concentrations in the presence of inhibitor **3** (three concentrations) and its absence are presented. The plots A and B show mixed-type inhibition.

The inhibitory mechanism of MB-gCs is demonstrated for compound **3** as an example. The graphical analysis using double reciprocal Lineweaver–Burk plots for compound **3** is shown in Fig. 2. The plots demonstrate that the binding of compound **3** to either AChE or BChE results in changes in *V*_max_ and *K*_m_. This suggests a mixed-type inhibition. The inhibition constant values were estimated as follows: for AChE, *K*_*i*_ = 0.88 ± 0.07 µM (competitive component), *αK*_*i*_ = 3.35 ± 0.31 µM (non-competitive component); and for BChE, *K*_*i*_ = 0.37 ± 0.03 µM, *αK*_*i*_ = 2.09 ± 0.19 µM. Similar results were obtained for AChE and BChE inhibition by compound **6** (AChE: *K*_*i*_ = 3.52 ± 0.29 µM, *αK*_*i*_ = 13.4 ± 1.2 µM; BChE: *K*_*i*_ = 0.53 ± 0.04 µM, *αK*_*i*_ = 2.64 ± 0.24 µM). Hence, the conjugates under investigation were found to be potent reversible mixed-type inhibitors of both cholinesterases.

### Molecular modeling of conjugates 3 and 6 and their interactions with human AChE and BChE

Estimates of p*K*_a_ values for the piperidine nitrogen of the gC ring of the considered compounds generated by ChemAxon and ACD were between 7 and 8. This allowed us to conclude that under our experimental conditions both protonated and non-protonated forms might be present. For this reason, both forms were used for molecular docking and considered for analysis of results.

Molecular docking into the human enzymes (AChE and BChE) showed that the positions of the compounds within the gorge of each protein depended markedly on the protonation state of the piperidine fragment. For both AChE and BChE, the protonated forms showed tighter binding and more specific interactions, although non-protonated forms also exhibited favorable binding.

**Figure 3:**
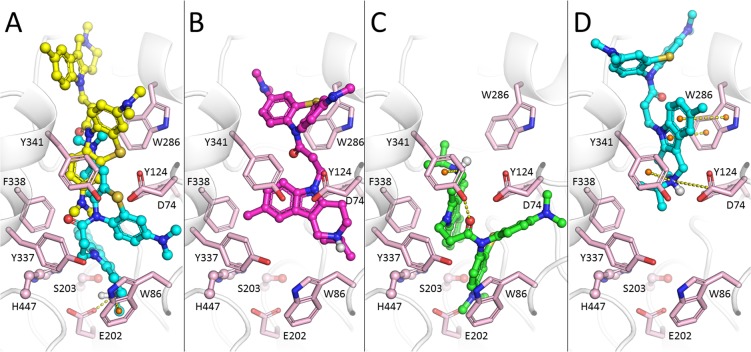
Positions of conjugates **3** and **6** in the gorge of AChE. (**A**) Overlay of positions of protonated form **3** (carbon atoms are colored cyan) in the CAS with the nonprotonated form (carbon atoms are colored yellow) in the PAS. (**B–D**) Various binding poses of the protonated form of conjugate **6**. (**B**) Above the CAS, (**C**) — in the CAS, (**D**) — in the PAS.

Conjugate **3** in its protonated form exhibited the most favorable pose in the catalytic active site (CAS) of AChE. Binding included an ion pair between the protonated nitrogen of the piperidine ring and Glu202 and π-cation interactions with Trp86. In contrast, the non-protonated form occupied only the PAS (Fig. 3A).

Conjugate **6** had a bulkier substituent at the nitrogen atom of the gC fragment (R^1^ = *i*-C_3_H_7_); due to this, the piperidine group was more distant from the AChE cation-binding site (Fig. 3B). There were two other major docking positions obtained: one with the MB group blocking the active site, stabilized by a hydrogen bond between carbonyl oxygen of the linker and Tyr341 side chain (Fig. 3C); and the other with the protonated piperidine group interacting with the PAS (Fig. 3D). Due to the bulkier *i*-C_3_H_7_ substituent, interactions with the AChE CAS of this molecular fragment were less tight than in the case of conjugate **3** (R^1^ = CH_3_). For the non-protonated form, the binding was mainly in the PAS. This docking result corresponded well with the experimentally observed weaker inhibitory activity toward AChE of conjugate **6** compared to **3** (Table 1).

**Figure 4:**
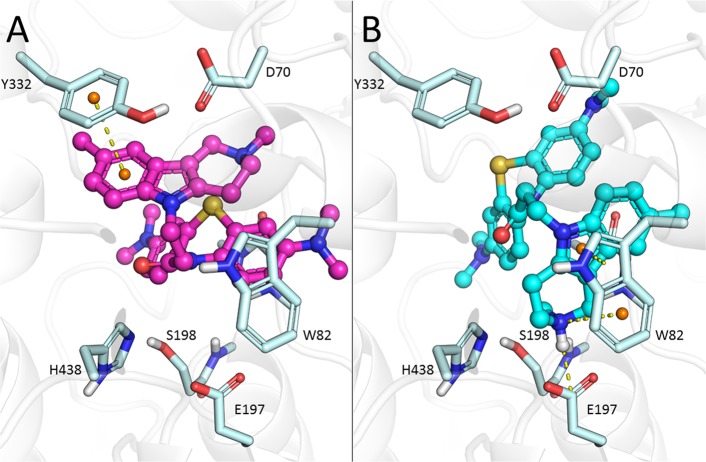
Docked positions of conjugate **3** in the gorge of BChE. (**A**) Non-protonated form occupying the PAS. (**B**) Protonated form in the CAS.

The gorge of BChE is considerably wider than that of AChE^65^. As in the case of AChE, the non-protonated form of conjugate **3** tended to bind to the PAS (Fig. 4A), but the piperidine fragment of the protonated form **3** was able to interact with the CAS (Fig. 4B). Similarly, conjugate **6**, with a bulkier substituent (R^1^ = *i*-C_3_H_7_), could be found in the CAS as well as in the PAS of BChE. Thus, molecular docking demonstrated that MB-gCs could bind both in the PAS and in the CAS of both cholinesterases, which is in agreement with their experimentally determined mixed-type inhibition. Compound **3** (R^1^ = CH_3_) fitted the CAS of AChE very tightly, and increasing the size of the R^1^-substituent (R^1^ = *i*-C_3_H_7_) led to the reduced binding affinity of compound **6**.

### Displacement of propidium iodide from the peripheral anionic site of *Ee*AChE by MB-gCs

The fluorescent assay used to evaluate competitive propidium iodide displacement from the PAS of AChE is commonly used as primary screening method of AChE pro-aggregation activity inhibitors. Propidium iodide is a selective ligand for the PAS of AChE responsible for Aβ binding. It exhibits a fluorescence increase upon binding to AChE. A decrease in propidium iodide fluorescence in the presence of test compounds suggests that they are able to displace propidium and can bind to the PAS of AChE. Here, donepezil and decamethonium were used as reference compounds.

All MB-gCs were examined for their ability to bind to the PAS of *Ee*AChE and competitively displace propidium iodide. The results are presented in Table 2. The compounds decreased fluorescence intensity by 6–14% at 3 µM, and by 29–37% at 20 µM. It should be noted that the conjugates displaced propidium from the PAS of AChE more effectively than the reference compounds donepezil and decamethonium. Their high activity is likely attributable to the MB moiety in their structures; MB on its own decreases fluorescence intensity by 20% at 3 µM concentration and by 40% at 20 µM. Our data suggest that the conjugates are able to bind to the PAS of AChE and therefore might be able to inhibit the aggregation of amyloid peptides induced by AChE.

**Table 2:** Inhibition of *Ee*AChE and displacement of propidium iodide from the PAS by MB-gCs.

Compound	*Ee*AChE IC_50_, µM	% Displacement of propidium iodide	
		3 µM	20 µM
**1**	0.83 ± 0.03	11.4 ± 0.6	32.5 ± 1.1
**2**	0.67 ± 0.04	12.9 ± 1.1	37.1 ± 2.0
**3**	1.47 ± 0.08	8.1 ± 0.7	35.6 ± 1.5
**4**	0.65 ± 0.05	11.8 ± 0.6	37.6 ± 1.8
**5**	0.80 ± 0.07	14.2 ± 0.9	35.1 ± 0.9
**6**	1.07 ± 0.08	7.7 ± 0.5	30.0 ± 0.9
**7**	2.09 ± 0.19	6.7 ± 0.5	29.8 ± 2.1
Donepezil	0.072 ± 0.007	9.4 ± 0.9	10.1 ± 0.6
Decamethonium	51.4 ± 2.2	3.5 ± 0.3	7.8 ± 0.6
Dimebon	32.4 ± 3.5	2.8 ± 0.2	6.0 ± 0.5
MB	0.21 ± 0.02	20.9 ± 1.9	40.8 ± 3.7
MBН_2_ (Leuco form)	0.28 ± 0.02	17.7 ± 1.8	36.9 ± 3.3

Data are mean ± SEM (*n* ≥ 3 experiments).

### Studies of radical-scavenging activity

Antioxidant activity was evaluated by employing two radical-scavenging assays: the ABTS and ORAC-FL tests.

#### Evaluation of MB-gCs for antiradical activity by the ABTS^·+^ cation-radical scavenging assay

This assay is based on the production of a stable dark green ABTS cation-radical (ABTS^**·+**^) by incubating ABTS ([2,2′-azino-bis(3-eth-ylbenzothiazoline-6-sulfonic acid diammonium salt)) with potassium persulfate. The subsequent interaction of ABTS^**·+**^ with an antioxidant compound causes a decrease in absorbance at 734 nm. The ABTS radical-scavenging activity of the compounds was measured according to an established method^66^ with some modifications, at 30 °С in the dark; incubation time was 1 hour. Concentration range for the conjugates was 1 × 10^–6^–2 × 10^–4^ М. Trolox was used as a reference antioxidant. The results were expressed as TEAC values (Trolox equivalent antioxidant capacity) calculated by dividing the slope of ABTS^**·+**^ concentration decrease versus the antioxidant concentration by the slope of the Trolox plot. For the most potent compounds IC_50_, μМ values were estimated (IC_50_ value is the concentration of the sample required to reduce the concentration of ABTS^**·+**^ by 50%). The lower the IC_50_, the more potently the compound scavenges the ABTS cation-radical. The results are presented in Table 3.

**Table 3:** Radical-scavenging activity of MB-gCs in the ABTS and ORAC-FL tests.

Compound	ABTS^·+^ scavenging activity		ORAC TE value (µmol Trolox/ µmol comp.)	HOMO-LUMO gap energy, eV
	TEAC value	IC_50_, μM		
**1**	0.63 ± 0.045	35.4 ± 3.4	7.28 ± 0.65	4.59
**2**	1.03 ± 0.07	19.1 ± 1.52	8.80 ± 0.96	4.65
**3**	0.79 ± 0.06	27.5 ± 1.64	7.44 ± 0.74	4.67
**4**	0.99 ± 0.08	20.5 ± 2.05	9.82 ± 0.79	4.62
**5**	0.99 ± 0.05	19.6 ± 1.82	7.70 ± 0.84	4.63
**6**	0.96 ± 0.07	21.2 ± 1.75	7.12 ± 0.64	4.63
**7**	1.08 ± 0.08	19.0 ± 2.28	10.93 ± 0.98	4.41
Dimebon	0.004	n.d.	1.07 ± 0.08	4.77
Trolox	1.0	20.4 ± 1.7	1.0	n.d.

Data are mean ± SEM, *n* = 3.

TEAC value = (Trolox equivalent antioxidant capacity) was determined from the ratio of the slopes of the concentration-response curves, test compound/Trolox.

n.d. = not determined.

The results showed that the MB-gCs have high ABTS^**·+**^ scavenging activity, close to or even greater than that of Trolox (Table 3). Moreover, all conjugates demonstrated a high initial reaction rate with the ABTS radical close to the rate for Trolox (data not shown). Dimebon did not show significant activity in the ABTS test.

#### Evaluation of antioxidant activity of conjugates of MB-gCs via the ORAC-FL method

The ability of the conjugates to reduce the amount of peroxyl radicals as another characteristic of the antioxidant activity of the compounds was determined using the oxygen radical absorbance capacity by fluorescence (ORAC-FL) method using fluorescein (FL) as a fluorescent probe. The method is based on measuring the decrease in the intensity of fluorescence with time, which characterizes the degree of decay of the fluorescent probe under the influence of peroxyl radicals. In the presence of antioxidants, the degree of decay of the fluorescent probe decreases and, accordingly, the fluorescence time increases.

The ability of compounds to scavenge peroxyl radicals was characterized by the value of the Trolox equivalent (TE, μmol of Trolox per μmol of the tested compound), which is equal to the ratio of the Trolox concentration to the tested compound concentration having the same fluorescence intensity. Peroxyl radical scavenging capacity of Trolox is taken as one (1)^67,68^. The data obtained in the ORAC-FL test are summarized in Table 3.

As observed in Table 3, MB-gCs possess a high peroxyl radical scavenging capacity, which exceeds that of Trolox and is in the range of 7 to 10 TE. In contrast, Dimebon did not show such a high anti-radical activity.

The results on radical-scavenging activity for MB and MBH_2_ are not shown in Table 3 because the activity for these compounds was not detectable with the ABTS and ORAC-FL methods. This negative result could be attributed to the very low redox potential of MB (11 mV)^69^ and consequently by its cycling between oxidized and reduced forms.

#### Frontier orbital calculations

Quantum chemical calculations for MB and its conjugates were performed using the DFT(B3LYP)/6-31 ++ G** method. The calculated HOMO-LUMO gap value of 2.275 eV for MB is in good agreement with literature data^70^ and confirms its extremely high reactivity and ease of redox cycling MBH_2_ ⇔ MB + 2 H. Higher energy values were obtained for the conjugates of MB with γ-carbolines (Table 3), which reflects stabilization of the MB molecule as a result of conjugation.

**Figure 5:**
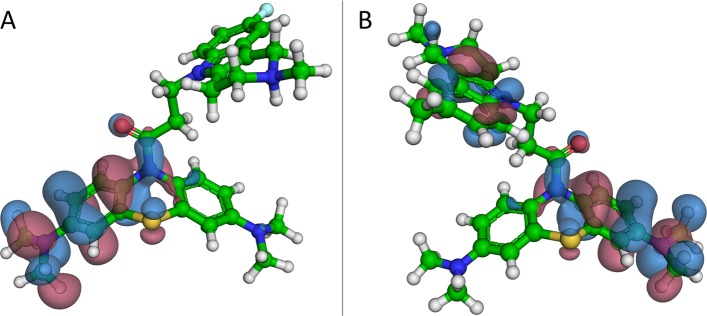
HOMO-orbitals for conjugates **7 (A)** and **3 (B)**.

The position of the HOMO orbitals in the conjugate molecules (Fig. 5**)** implies that the scavenging of free radicals is carried out by the MB fragment, presumably by its sulfur atom. This interpretation is supported by the strong aromatic conjugation of the amide group in the attachment region of the MB moiety (the configuration is almost planar–the dihedral angle is 11°) and the notably lower aromatic conjugation of the sulfur atom (the dihedral angle is 37°)^24^. The HOMO orbitals are predominantly localized on half of the symmetrical fragment of MB. Moreover, they depend on the orientation of the γ-carboline fragment, so that the energy levels of the orbitals in different conformers are almost the same.

### Interaction of MB-gCs with mitochondria

#### Inhibition of iron-induced LP in isolated rat liver mitochondria

We tested the compounds against lipid peroxidation (LP) induced by Fe^3+^ ions in isolated rat liver mitochondria. All the conjugates as well as MB and its reduced form MBH_2_ effectively blocked iron-induced LP in mitochondria (Table 4**)**. IC_50_ values of the tested compounds were in the micromolar or submicromolar range. On the other hand, Dimebon did not inhibit iron-induced LP in mitochondria at a concentration of 30 µM. Thus, these results allowed us to suggest that the mechanisms of antioxidant action of the conjugates may be connected with their redox-cycling properties, in some degree similar to those of MB (MBH_2_), and perhaps manifested in different stages of the complex chain of events in lipid peroxidation.

**Table 4:** Effect of MB-gCs on mitochondrial characteristics and inhibition of Fe^3+^-induced LP.

Compound	ΔΨm, %		IC_50_ of Fe^3+^-induced LP, μM
	CI (g/m)	CII (s/r)	
**1**	20 ± 1	64 ± 2	1.45 ± 0.29
**2**	7 ± 1	48 ± 1	0.68 ± 0.07
**3**	29 ± 11	67 ± 2	2.29 ± 0.47
**4**	9 ± 1	43 ± 3	0.71 ± 0.01
**5**	17 ± 2	69 ± 2	0.75 ± 0.01
**6**	8 ± 2	44 ± 5	0.58 ± 0.03
**7**	13 ± 3	67 ± 1	3.20 ± 0.40
MB	14 ± 7	40 ± 9	4.0 ± 0.6
MBH_2_	0	18 ± 1	1.58 ± 0.49
Dimebon	0	0	> 30

ΔΨm data are mean values normalized to the control probe ± SD, *n* ≥ 5 experiments.

ΔΨm** = **% depolarization of mitochondrial membrane potential after 10-min incubation with 30 µМ of compounds for the following conditions:

CI (g/m): energized with Complex-I substrates (glutamate, malate);

CII (s/r): energied with a Complex-II substrate (succinate) in the presence of the Complex-I inhibitor, rotenone.

Lipid peroxidation (LP) was induced by 0.5 mM Fe^3+^.

IC_50_ values are mean ± SEM, n ≥ 3 experiments.

#### Action of MB-gCs on transmembrane potential of isolated rat liver mitochondria

Mitochondria and the mitochondrial permeability transition (MPT) are key players in the cascades of events leading to cell death^71^. Consequently, inhibition of the MPT is a promising target in neuroprotection^3^. On the other hand, depolarization of mitochondria is widely used a predictor of toxicity^72^ but depolarization connected with uncoupling of the respiratory chain or stimulation of electron flux can also be cytoprotective^73^. Due to its redox-cycling capability, MB can restore the electron flux in the respiratory chain in the presence of inhibitors of complex-I^74^. Furthermore, γ-carbolines, such as Dimebon, also exhibit neuroprotection, which is thought to involve inhibition of the MPT^75^. Consequently, we measured the effect of our conjugates of MB and γ-carbolines on mitochondrial membrane potential under two conditions. (C1) Mitochondria were energized with NADH-dependent substrates of Complex-I (glutamate and malate). (CII) Mitochondria were energized with an FADH_2_-dependent substrate of Complex II (succinate) in the presence of rotenone, a Complex-I inhibitor. Conjugates (30 μM) were incubated with mitochondria and the effect on mitochondrial membrane potential was measured. For comparison, mitochondria were incubated with MB, MBH_2_, and Dimebon (30 μM each).

The data presented in Table 4 show that Dimebon had no effect on mitochondrial membrane potential, either in the presence of NADH-dependent substrates, or in the presence of FADH_2_-dependent substrates. MB slightly depolarized (14 ± 7%) mitochondria in the presence of glutamate and malate, and depolarized to a greater extent in the presence of succinate and rotenone (40 ± 9%). MBH_2_, the reduced form of MB, was less active in this test: there was no depolarization after 10 min incubation in the presence of Complex-I substrates, and a slight depolarization in the presence of a Complex-II substrate (18 ± 1%). The tested conjugates had a similar effect on the mitochondrial membrane potential as MB: i.e., all compounds induced some degree of depolarization. For some compounds (**1**, **3**, **5**) their depolarizing activity at 30 μM surpassed that of MB in the presence of FADH_2_-dependent substrates. Whereas depolarization was detectable in condition CI and readily apparent in condition CII for 30 μM concentrations of compounds, depolarization was undetectable in both conditions for compounds incubated at 3 μM (data not shown).

**Figure 6:**
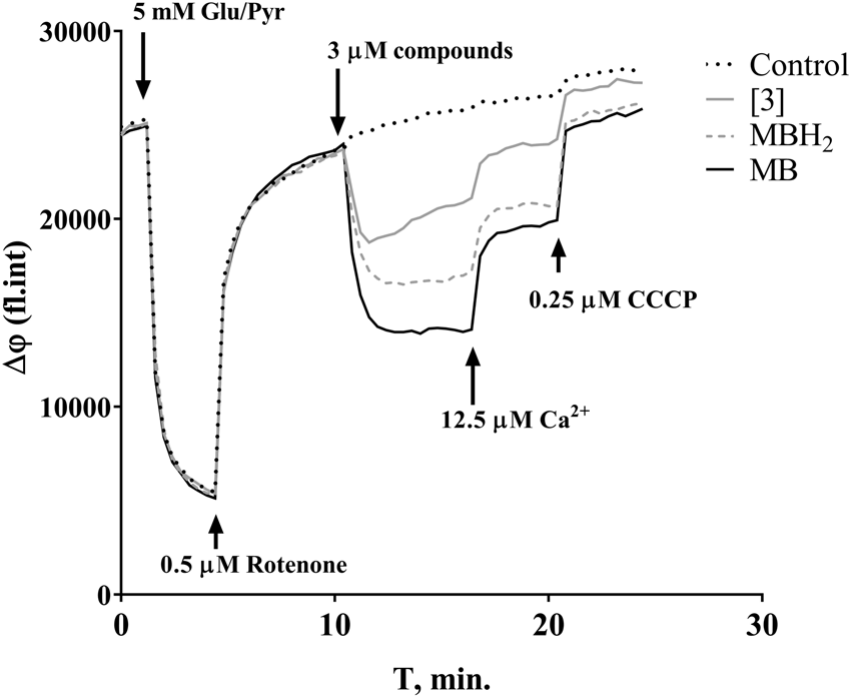
Restoration of the mitochondrial membrane potential by compound **3**, MB, and MBH2 after rotenone-induced depolarization.

Although it appeared promising that the conjugates exhibited antioxidant capability and affected mitochondrial membrane potential as observed for MB, it is possible that the depolarization produced by the conjugates was deleterious rather than protective. Therefore, we examined the ability of MB, MBH2, and compound **3** to restore membrane potential after depolarization with rotenone. As shown in Fig. 6, rotenone – an inhibitor of Complex-I of the respiratory chain – induced an abrupt drop in ΔΨm of glutamate/pyruvate-supported mitochondria, which recovered after addition of 3 μM MB, MBH_2_ and conjugate **3**. It is important to note that successive additions of calcium and the uncoupler carbonyl cyanide m-chlorophenyl hydrazone (CCCP) each caused mitochondrial depolarization (Fig. 6). Thus, these results indicate that conjugate **3** acts similarly to MB and MBH_2_ on mitochondrial membrane potential and support our hypothesis of a possible redox-cycling mechanism for our conjugates.

## Discussion

Two enzymes hydrolyzing the neurotransmitter acetylcholine — AChE and BChE, play important roles in the clinical course and pathogenesis of AD and AD-type dementia. AChE inhibitors aim to compensate for the deficit in acetylcholine^76^. In healthy brain, acetylcholine is predominantly (80%) hydrolyzed by AChE, whereas BChE plays a supplementary role. However, as AD progresses, AChE activity decreases, but BChE activity gradually increases. Consequently, the significance of BChE as a therapeutic target for reducing the cholinergic deficiency in AD is being increasingly recognized^77,78^. Drugs inhibiting both cholinesterases (AChE and BChE) are assumed to enhance treatment efficacy^79^. The enzyme CaE, structurally related to cholinesterases, hydrolyses numerous pharmaceuticals containing ester groups^80^. Therefore, CaE inhibition by anticholinesterase drugs may induce undesirable drug-drug interactions^63,81^. Thus, the esterase profile approach, employed here, i.e., the comparative evaluation of a compound’s inhibitory activity against several esterases^63,64,82,83^, contributes to the early detection of possible adverse effects connected with CaE inhibition.

The data given in Table 1 show that all tested conjugates demonstrate low inhibitory activity toward CaE and rather high activity against of AChE and BChE, with IC_50_ values in the micromolar and submicromolar range. Unlike previously reported conjugates of phenothiazine and γ-carbolines^24^, which selectively inhibited BChE, conjugates of MB with γ-carbolines do not have a clear selectivity for cholinesterases. The conjugates MB-gCs inhibit AChE and BChE to the same or even higher extent than their component pharmacophores. Compounds **3** (R = R^1^ = CH_3_) and **6** (R = CH_3_, R^1^ = *i*-C_3_H_7_) exhibited the maximum activity toward both cholinesterases.

AChE and BChE inhibition by the conjugates MB-gCs is reversible and has a mixed-type mechanism of action (Fig. 2**)**. This is in complete agreement with molecular docking results, which revealed that these conjugates could bind to both the CAS and PAS of AChE and BChE. Compound **3** (R^1^ = CH_3_) fits the CAS of AChE very tightly, and increasing the size of the R^1^ substituent (compound **6**, R^1^ = *i*-C_3_H_7_) leads to reduced binding affinity, which is also observed experimentally (Table 1). The binding of conjugates to BChE, which has a larger active site, is less sensitive to substituent R^1^ size. This is in a good agreement with experimental results for compounds **3** and **6**.

It has been shown that AChE promotes the formation of Aβ fibrils *in vitro* and Aβ plaques in the cerebral cortex of transgenic murine models of AD^84^. АChE plays a pivotal role in the processing of β-amyloid plaques by means of the PAS, which interacts with soluble β-amyloid peptides promoting their aggregation^9,10,85,86^. The structural motif of AChE that promotes β-amyloid peptide fibril formation is located in the PAS, and Trp279 (*Torpedo californica* numbering) plays an important role in this process^9^. This hypothesis has strong evidence. It is supported by studies demonstrating that ligands binding selectively to the PAS, such as propidium iodide, are capable of blocking Aβ aggregation^85^. A recent study of a transgenic APP/PS1 murine model showed that ligands of the AChE PAS not only improve memory, but also lead to significant decreases in the area and number of β-amyloid peptide plaques in the brain^87^. Therefore, the development of drugs blocking the PAS of AChE and affecting its interactions with β-amyloid peptide (and thus decreasing its AChE-induced aggregation) is a promising approach for anti-amyloid AD treatment.

We have shown that conjugates of MB with γ-carbolines are mixed-type AChE inhibitors, and according to molecular docking data can bind to the PAS of AChE. Figure 3 demonstrates that the tested compounds form π-cation interactions with Trp286 of human AChE, which corresponds to Trp279 in the PAS of *Torpedo californica* AChE^9^. The experimental decrease in fluorescence intensity of propidium iodide bound to AChE in the presence of our conjugates confirms their binding to the PAS of AChE by displacement of propidium. Taken together, our data support the rationale of using such conjugates as a starting point for the development of new anti-amyloid drugs.

The total primary antioxidant capacity of our conjugates was evaluated using two tests: ABTS and ORAC. In both tests, the conjugates demonstrated high activity. The ABTS method demonstrated that all of the novel conjugates MB-gCs have radical-scavenging activity, equal to that of Trolox (Table 3). Moreover, the high initial reaction rate of the conjugates with the ABTS radical supports the SET (Single Electron Transfer) mechanism of anti-radical activity^88,89^. The complementary ORAC test characterizes the ability of compounds to scavenge the more reactive peroxyl radical, which mimics lipid peroxyl radicals involved in the lipid peroxidation chain reaction *in vivo*. In this test, the activity of the novel conjugates exceeded that of Trolox by 7- to 10-fold (Table 3).

Quantum chemical DFT calculations for MB and its conjugates with γ-carbolines supported the experimental data on the primary antioxidant activity of the conjugates. The HOMO-LUMO energy gap value of 2.275 eV for MB, is in good agreement with its extremely high reactivity and ease of redox cycling for the reaction MBH_2_ ⇔ MB + 2 H. Conjugation of MB with γ-carbolines increases the stability of the molecules as evidenced by higher HOMO-LUMO gap energy values exceeding 4 eV. Furthermore, the position of the HOMO orbitals in the conjugates (Fig. 5**)** implies that free radical scavenging is carried out by the MB fragment, presumably by its sulfur atom.

We have previously reported that Dimebon blocked spontaneous, tBHP- and β-amyloid-induced LP in mitochondria^60^. However, in concentrations less than 30 µM, Dimebon has almost no impact on Fe^3+^-induced LP in mitochondria, whereas the novel conjugates MB-gCs are effective antioxidants (Table 4). IC_50_ values lie in the range 0.5 to 4 µM (Table 4**)**. MB and MBH_2_ have similar antioxidant activity, suggesting that the ability of conjugates MB-gCs to block Fe^3+^-induced LP could be attributed to the phenothiazine moiety of MB and its redox-cycling properties.

There are two possible mechanisms of LP inhibition by MB and its γ-carboline derivatives MB-gCs. First, these compounds might interact with free radicals and terminate chain reactions owing to their redox-cycling properties. The other possible mechanism is a decrease in ROS production by mitochondria owing to mitochondrial depolarization. It was previously reported that MB depolarizes mitochondria because of its capacity for alternative mitochondrial electron transfer^90^. Its antioxidant and neuroprotective effects could be also attributed to that ability. Our data showed that the novel conjugates of MB and γ-carbolines can also depolarize mitochondria and hence could decrease calcium-induced mitochondrial permeability transition. In addition, we have shown that a representative of the MB-γ-carboline conjugates (**3**) exhibits the important property of being able to restore the mitochondrial membrane potential in the presence of rotenone, an inhibitor of Complex-I.

## Conclusions

We found that novel conjugates of MB with γ-carbonline derivatives effectively inhibit AChE and BChE with IC_50_ values of 1–10 µM. At the same time, they exhibited very low potencies against CaE, thus precluding potential drug-drug interactions arising from CaE inhibition. Kinetic studies showed that the conjugates were mixed-type reversible inhibitors of both cholinesterases. Molecular docking results indicated that the compounds bind both to the CAS and PAS of AChE and BChE. Binding of conjugates to the PAS of AChE along with mixed type AChE inhibition suggest their potential to block AChE-induced aggregation of β-amyloid. Indeed, the compounds studied effectively displaced propidium from the PAS of AChE (30–37% at 20 µM). Additionally, conjugates were extremely active in both radical-scavenging tests. In this regard, their activity was comparable with that of Trolox in the ABTS test (TEAC = 0.96–1.08), while their ability to scavenge peroxyl radicals determined by the ORAC-FL method considerably exceeded Trolox and ranged from 7 to 10 TE. Quantum mechanical DFT calculations suggest that free radical scavenging is mediated by the MB fragment, presumably via its sulfur atom. Finally, the conjugates effectively prevent lipid peroxidation of mitochondria, and a representative compound at 3 μM concentration was able to restore the mitochondrial membrane potential after its depolarization by the Complex-I inhibitor, rotenone. These results allow us to suggest that the conjugates possess redox-cycling properties that are similar to some degree to that of MB. Overall, the conjugates have exhibited favorable properties in all of the experimental and computational determinations, thus suggesting that they are promising candidates for the development of multitarget disease-modifying drugs for treating AD and related neuronal pathologies.

## Experimental

### *In vitro* AChE, BChE, and CaE inhibition

Acetylcholinesterase (AChE, EC 3.1.1.7, from human erythrocyte and *E*. *electricus* (*Ee*AChE) (type VI-S), butyrylcholinesterase (BChE, EC 3.1.1.8, from equine serum), carboxylesterase (CaE, EC 3.1.1.1, from porcine liver), acetylthiocholine iodide (ATCh), butylthiocholine iodide (BTCh), 5,5′-dithiobis-(2-nitrobenzoic acid) (DTNB), 4-nitrophenyl acetate (4-NPA), were purchased from Sigma-Aldrich (Germany).

AChE and BChE activities were measured by the Ellman method as described earlier^91^. The assay solution consisted of 0.1 M K/Na phosphate buffer pH 7.5, 25 °C with the addition of 0.33 mM DTNB, 0.02 unit/mL of AChE or BChE, and 1 mM of substrate (ATCh or BTCh, respectively). The assays were carried out with a reagent blank containing all components except AChE or BChE to account for non-enzymatic hydrolysis of substrate. In addition, an enzyme blank was included that contained all components except substrate to account for non-substrate sulfhydryl groups. The activity of CaE was determined spectrophotometrically by the release of 4-nitrophenol at 405 nm^92^. The assay solution consisted of 0.1 M K/Na phosphate buffer pH 8.0, 25 °C with the addition of 1 mM 4-nitrophenyl acetate and 0.02 unit/mL of CaE. Assays were carried out with a blank containing all components except CaE.

The test compounds were dissolved in DMSO; the incubation mixture contained 2% (v/v) of the solvent. Eight different concentrations of the test compounds in the range 10^−11^–10^−4^ M were selected in order to obtain inhibition of AChE and BChE activity between 20% and 80%. The test compounds were added to the assay solution and preincubated at 25°C with the enzymes for 5 min followed by the addition of substrate. A parallel control was made for the assay solution with no inhibitor. Measurements were performed in a FLUOStar OPTIMA microplate reader (BMG Labtech, Germany). Each experiment was performed three times. The results were expressed as the mean ± SEM. The reaction rates in the presence and absence of inhibitor were compared, and the percent of residual enzyme activity due to the presence of test compounds was calculated. IC_50_ values were determined graphically from inhibition curves using Origin 6.1 for Windows (OriginLab, Northampton, MA).

### Kinetic analysis of AChE and BChE inhibition. Determination of steady state inhibition constants

To elucidate the inhibition mechanisms for the active compounds, the AChE and BChE residual activity was determined in the presence of 3 increasing concentrations of the test compounds and 6 decreasing concentrations of the substrates. The test compounds were preincubated with the enzymes at 25 °C for 5 min, followed by the addition of the substrates. Parallel controls were made for an assay of the rate of hydrolysis of the same concentrations of substrates in the solutions with no inhibitor. Measurements were performed in a FLUOStar OPTIMA microplate reader (BMG Labtech, Germany). Each experiment was performed three times. Results were fitted into Lineweaver-Burk double-reciprocal kinetic plots of 1/V versus 1/[S] and values of inhibition constants *K*_*i*_ (competitive component) and *αK*_*i*_ (noncompetitive component) were calculated using Origin 6.1 for Windows (OriginLab, Northampton, MA).

### Propidium displacement studies

The ability of the test compounds to competitively displace propidium iodide, a selective ligand of the PAS of AChE, was evaluated by a fluorescence method^93,94^. *Ee*AChE was used owing to its high degree of purification, high activity, and lower cost than human AChE. In addition, we performed a 3D alignment of the crystal structures of *Ee*AChE (PDB 1C2O) and human AChE (PDB 4EY7) using YASARA-Structure 18.4.24 for Windows, which showed that the two structures were essentially congruent with an RMSD of 0.623 Å over 527 aligned residues and 88.6% sequence identity. The fluorescence intensity of propidium iodide bound with AChE increases several times. The decrease of fluorescence intensity of propidium iodide in the presence of the test compounds shows their ability to bind to the peripheral anionic site of AChE, which predicts that the compounds would block the AChE-mediated aggregation of β-amyloid.

To determine the degree of displacement (% displacement) of propidium iodide from the PAS of AChE, *Ee*AChE (final concentration 7 μM) was incubated with the test compound at a concentration of 3 and 20 μM in 1 mM Tris-HCl buffer pH 8.0, 25 °C for 15 min. Then, propidium iodide solution (final concentration 8 μM) was added, the samples were incubated for 15 min and the fluorescence spectrum (530 nm (excitation) and 600 nm (emission)) was taken. Donepezil and tacrine were used as reference compounds. The blank contained propidium iodide of the same concentration in 1 mM Tris-HCl buffer pH 8.0. The measurements were carried out in triplicate on a microplate reader FLUOStar Optima (BMG Labtech Germany).

The degree of displacement (% displacement) of propidium iodide from the peripheral anionic site of AChE was calculated by the following formula:

$$ \% \,{\rm{Displacement}}=100-({{\rm{IF}}}_{{\rm{AChE}}+{\rm{Propidium}}+{\rm{inhibitor}}}{/\mathrm{IF}}_{\mathrm{AChE}+\mathrm{Propidium}})\,-\,100,$$

where IF_AChE + Propidium_ is the fluorescence intensity of the propidium associated with AChE in the absence of the test compound (taken as 100%), and IF_AChE + Propidium + inhibitor_ is the fluorescence intensity of the propidium associated with AChE in the presence of the test compound.

### ABTS radical cation scavenging assay

Radical scavenging activity of the compounds was assessed using an ABTS radical decolorization assay^66^ with some minor modifications. ABTS (2,2′-azino-bis-(3-ethylbenzothiazoline-6-sulfonic acid) diammonium salt) was purchased from TCI (Tokyo, Japan); potassium persulfate (di-potassium peroxodisulfate) and Trolox (6-hydroxy-2,5,7,8-tetramethychroman-2-carboxylic acid were obtained from Sigma-Aldrich Chemical Co. (St. Louis, MO, USA). Ethanol was HPLC grade. Aqueous solutions were prepared using deionized water.

Trolox was used as the antioxidant standard. A 5 mM solution of Trolox was prepared in DMSO for use as stock. Fresh working solutions of known concentrations (1–100 μM) were prepared on the day of experiments and used for calibration and as positive controls for ABTS radical cation (ABTS^•+^) scavenging activity. ABTS was dissolved in deionized water to a 7 mM concentration. The solution of ABTS^•+^ was produced by mixing 7 mM ABTS stock solution with 2.45 mM potassium persulfate aqueous solution in equal quantities and allowing them to react for 12–16 h at room temperature in the dark. At the time of activity measurement, ABTS^•+^ solution was diluted with ethanol to adjust to an absorbance value of about 0.80 ± 0.02 at 734 nm. Fresh working ABTS^•+^ solution was prepared for each assay.

The radical scavenging capacity of the compounds was analyzed by mixing 10 μl of compound with 240 μl of ABTS^•**+**^ working solution. The reduction in absorbance was measured spectrophotometrically at 734 nm after 1 h of mixing the solutions using the microplate UV/VIS spectrophotometer BioRad xMark (Japan). Ethanol blanks were run in each assay. Values were obtained from three replicates of each sample and three independent experiments.

Antioxidant capacity as a Trolox equivalent (TEAC values) was determined as the ratio between the slopes obtained from the linear correlation for concentrations of test compounds and Trolox with absorbance of ABTS radical. For the most active compounds, we also determined the IC_50_ values (compound concentration required for 50% reduction of ABTS radical). The compounds were tested in the concentration range of 1 × 10^−6^–1 × 10^−4^ M. The IC_50_ values were calculated using Origin 6.1 for Windows (OriginLab, Northampton, MA).

### Oxygen radical absorbance capacity assay

The ORAC-FL method of Ou *et al*.^67^, partially modified by Dávalos *et al*.^68^ was followed, using a FLUOStar Optima microplate reader (BMG Labtech, Germany) with 485-P excitation and 520-P emission filters. 2,2′-Azobis-(amidinopropane) dihydrochloride (AAPH), (±)-6-hydroxy-2,5,7,8-tetramethylchromane-2-carboxylic acid (Trolox) and fluorescein (FL) were purchased from Sigma-Aldrich. The reaction was carried out at 37 °C in 75 mM K,Na phosphate buffer (pH 7.4), and the final reaction mixture was 200 µL. The tested compounds and Trolox standard were dissolved in DMSO to 10 mM and further diluted in 75 mM K,Na phosphate buffer (pH 7.4). The final concentrations were 0.1–1 µM for the test compounds and 1–6 µM for Trolox. The blank was composed of 20 µL 75 mM K,Na phosphate buffer (pH 7.4) containing 2% (v/v) DMSO, 120 µL FL and 60 µL AAPH, and was added in each assay. Antioxidant (the test compound or Trolox, 20 µL) and FL (120 µL, final concentration: 70 nM) solutions were placed in a black 96-well microplate and were pre-incubated for 15 min at 37 °C. AAPH solution (60 µL, final concentration 12 mM) was then added rapidly using a multichannel pipette. The fluorescence was recorded every minute for 100 min. A Trolox standard curve was also obtained in each assay. All reactions were carried out in triplicate and at least three different assays were performed for each sample.

Antioxidant curves (fluorescence vs. time) were first normalized to the curve of the blank (without antioxidant) corresponding to the same assay, and the area under the fluorescence decay curve (AUC) was calculated. The net AUC corresponding to a sample was calculated by subtracting the AUC corresponding to the blank. Regression equations were calculated by plotting the net AUC against the antioxidant concentration. The ORAC value was obtained by dividing the slope of the latter curve by the slope of the Trolox curve obtained in the same assay. Final ORAC values were expressed as µmol Trolox per µmol test compounds where the value of Trolox was taken as 1. Data were expressed as means ± SEM.

### Rat liver mitochondria isolation

All experiments with animals were in compliance with the Guidelines for Animal Experiments at the Institute of Physiologically Active Compounds of the Russian Academy of Science (IPAC RAS). Rat liver mitochondria were isolated from Wistar strain male rats aged 3.5–4 months old (250–350 g). The rats were fasted overnight, then anesthetized by carbon dioxide and decapitated using a guillotine. The liver was quickly removed and homogenized in an ice-cold isolation buffer (225 mM mannitol, 75 mM sucrose, 5 mM HEPES, 1 mM EGTA, pH 7.6). Then rat liver mitochondria were isolated by conventional differential centrifugation^75^. The mitochondrial protein concentration was determined using a biuret procedure with bovine serum albumin as the standard^95^.

### Mitochondrial membrane potential

Safranine O (10 µM) was used as a membrane potential probe^96^. Fluorescence intensity at 580 nm (excitation at 520 nm) was measured with Victor3 multi-well fluorescence plate reader (Perkin Elmer, Germany). Mitochondrial protein concentration was 0.2 mg/ml. The medium for measurements contained 75 mM sucrose, 225 mM mannitol, 10 mM K-HEPES, 0.02 mM EGTA, and 1 mM KH_2_PO_4_ (pH 7.4, 25 °C). After a 5-min incubation, 5 mM glutamate/malate or 5 mM succinate and 0.5 μM rotenone were added to produce the mitochondrial potential. Then the compounds (30 μM) or the same volume of vehicle (DMSO) were injected into the mitochondrial suspension. After 15–20 min, CaCl_2_ (12.5 μM) was added to each probe to induce the depolarization of mitochondria and after 5 min 0.5 µM carbonyl cyanide m-chlorophenyl hydrazone (CCCP) was added for maximum depolarization of mitochondria. The level of depolarization **(ΔΨm)** was calculated from the fluorescence value after a 10-min incubation with 30 µМ of compounds (or vehicle) normalized between fluorescence measurements after substrate and CCCP additions.

For the determination of a compound’s ability to recover mitochondrial potential after rotenone depolarization, glutamate/malate (5 mM final concentration) and rotenone (0.5 μM final concentration) were added to the mitochondrial suspension followed by addition of the study compound.

### Mitochondrial lipid peroxidation

Lipid peroxidation in mitochondrial suspension was followed by the accumulation of substances that reacted with thiobarbituric acid (TBARs), and monitored spectrophotometrically according to the procedure described earlier^97^. Briefly, the experiments were carried out at 30 °C for 40 min in 0.25 ml of the reaction medium containing 125 mM sucrose, 65 mM KCl, 10 mM Hepes buffer (pH 7.4), 5 μM rotenone, 5 mM succinate and mitochondria (2 mg of protein·ml^−1^) in the presence or absence of study compounds or vehicle (DMSO). The final concentration of DMSO in the suspension was 0.2% (v/v), a concentration that did not show appreciable interference with the reaction as evidenced by control experiments. Oxidative damage to mitochondrial membranes (lipid peroxidation, LP) was induced by using FeNH_4_(SO_4_)_2_·12H_2_O (Fe^3+^; 0.5 mM) as the oxidizing agent.

### Mitochondrial statistical analyses

All experiments with mitochondria were repeated in at least five separate preparations. Results on mitochondrial membrane potential were presented as the normalized mean ± SD for mitochondrial potential measurements. The IC_50_ values for antioxidant activities of compounds were calculated using Origin 6.1 for Windows (OriginLab, Northampton, MA) and presented as mean ± SEM.

### Molecular modeling

The γ-carboline part of the compounds under consideration contains a piperidine ring condensed with an aromatic system that implicates the existence of conformers and enantiomers. Using OpenEye software^98^ (OMEGA 2.5.1.4: OpenEye Scientific Software, Santa Fe, NM. http://www.eyesopen.com) four configurations of the piperidine ring were generated. Estimates of p*K*a values were generated with Marvin 14.9.1.0 (ChemAxon, http://www.chemaxon.com) and ACD software, using both the Classic and GALAS (Global, Adjusted Locally According to Similarity) algorithms (ACD Labs Percepta p*K*a module, version 2016.2, Advanced Chemistry Development, Inc., Toronto, On, Canada, www.acdlabs.com, 2017). Geometries of the generated structures, with neutral and protonated piperidine rings, were quantum-mechanically optimized with Gamess-US^99^ software (B3LYP/6-31 G*). Frontier orbitals energies were calculated with the B3LYP/6-311 ++ G** level of theory. For molecular docking, the optimized structures of the ligands were used with partial atomic charges derived from QM results according to the Löwdin scheme^100^.

For human AChE, the X-ray structure PDB ID 4EY7 (hAChE co-crystallized with Donepezil, 2.35 Å^101^) was used due to its favorable resolution (2.35 Å) and established docking results with bulky inhibitiors^87^.

The X-ray structure of human BChE (hBChE, PDB ID 1P0I^102^) was used. Previously the importance of saturation of the BChE gorge with water molecules was demonstrated^103^. The protein structure was prepared, saturated with water molecules, and optimized using a QM/MM method as reported previously^103,104^. Molecular docking with a Lamarckian Genetic Algorithm (LGA)^105^ was performed with Autodock 4.2.6 software^106^. The grid box for docking included the whole active site gorge of AChE (22.5 Å × 22.5 Å × 22.5 Å grid box dimensions) and BChE (15 Å × 20.25 Å × 18 Å grid box dimensions) with a grid spacing of 0.375 Å. The main LGA parameters were 256 runs, 25 × 10^6^ evaluations, 27 × 10^4^ generations and a population size of 300. Structural images were prepared with PyMOL (Schrödinger, LLC). Calculations were performed at the Lomonosov-2 supercomputer^107^.
